# Evaluating 3D Hand Scanning Accuracy Across Trained and Untrained Students

**DOI:** 10.3390/bioengineering12070777

**Published:** 2025-07-18

**Authors:** Ciprian Glazer, Mihaela Oravitan, Corina Pantea, Bogdan Almajan-Guta, Nicolae-Adrian Jurjiu, Mihai Petru Marghitas, Claudiu Avram, Alexandra Mihaela Stanila

**Affiliations:** 1 Faculty of Physical Education and Sport, West University of Timisoara, 300223 Timisoara, Romania; ciprian.glazer@e-uvt.ro (C.G.); alexandra.rusu@e-uvt.ro (A.M.S.); 2 Department of Mechanics and Strength of Materials, Politehnica University of Timisoara, 300006 Timisoara, Romania; 3 National Institute of Research and Development for Electrochemistry and Condensed Matter (INCEMC), 300569 Timisoara, Romania; 4 Faculty of Medicine, “Victor Babeș” University of Medicine and Pharmacy Timisoara, 300041 Timisoara, Romania

**Keywords:** 3D scanning, 3D modeling, hand geometry, medical applications

## Abstract

*Background and Objectives:* Three-dimensional (3D) scanning is increasingly utilized in medical practice, from orthotics to surgical planning. However, traditional hand measurement techniques remain inconsistent and prone to human error and are often time-consuming. This research evaluates the practicality of a commercial 3D scanning method by comparing the accuracy of scans conducted by two user groups. *Materials and Methods:* This study evaluated the following two groups: an experimental group (n = 45) and a control group (n = 42). A total of 261 hand scans were captured using the Structure Sensor Pro 3D scanner for iPad (Structure, Boulder, CO, USA). The scans were then evaluated using Meshmixer software (version 3.5.474), analyzing key parameters, such as surface area, volume, number of vertices, and triangles, etc. Furthermore, a digital literacy test and a user experience survey were conducted to support a more comprehensive evaluation of participant performance within the study. *Results:* The experimental group outperformed the control group on all measured parameters, including surface area, volume, vertices, triangle, and gap count, with large effect sizes observed. User experience data revealed that participants in the experimental group rated the 3D scanner significantly higher across all dimensions, particularly in ease of use, excitement, supportiveness, and practicality. *Conclusions:* A short 15 min training session can promote scan reliability, demonstrating that even minimal instruction improves users’ proficiency in 3D scanning, fundamental for supporting clinical accuracy in diagnosis, surgical planning, and personalized device manufacturing

## 1. Introduction

In recent years, 3D scanning has gained more attention in medicine, improving diagnostic workflows and enabling personalized treatment planning, particularly in orthotics and prosthetics. By generating 3D models of the body’s structures, 3D scanning provides a faster and more accurate alternative for visualizing anatomical structures compared to traditional imaging methods. Its applications cover multiple disciplines, including orthopedics, medical device fabrication, and even medical teaching materials. Compared to conventional anthropometric measurements, 3D scanning provides a more dynamic and comprehensive visualization of patient anatomy [1,2]. Obtaining accurate anatomical measurements is essential in medical practice and research. However, traditional methods, such as manual anthropometry and caliper-based techniques, often fall short due to human error and the time-consuming nature of the process [3,4]. In contrast, 3D scanning offers a faster, non-invasive, and more reliable alternative, reducing the chance of inconsistencies, while improving accuracy and efficiency. Commonly used technologies for hand scanning include structured light, laser scanning, and photogrammetry. Structured light is widely adopted for its high-resolution capabilities and speed, while laser scanners offer superior accuracy across varied surfaces but are typically more expensive and less portable. Photogrammetry, although more affordable, is more susceptible to variability due to lighting and user technique [3,5]. Through advancements in structured-light and laser scanning technology, 3D scanners are strengthening their role in medical applications [5,6].

Scanning the human hand remains one of the most complex applications of 3D imaging due to its intricate musculoskeletal structure, individual anatomical variations, and frequent involuntary micro-movements. These factors complicate both the acquisition and the consistency of 3D data, making the hand one of the most difficult body parts to scan with accuracy [2,5]. Achieving precision and reliability in these scans requires advanced techniques and specialized equipment to capture the details of hand geometry. This need is particularly evident in developing medical devices, including orthotics, prosthetics, ergonomic tools, and rehabilitation monitoring systems, where even minor measurement deviations can affect functionality, patient comfort, and clinical outcomes [1,2,7,8].

Training programs and access to appropriate equipment are essential for effectively implementing 3D scanning technologies. Several studies underline that user expertise significantly influences scan quality. Even with advanced scanning tools, untrained operators may produce inconsistent results. Therefore, structured training protocols are increasingly seen as essential, especially in clinical or educational settings where non-specialists perform the scans [2,3]. Such programs would provide healthcare professionals with the necessary skills to perform and interpret scans within medical practice, eliminating the need for advanced expertise in imaging or engineering [3]. Moreover, the availability of cost-effective solutions could facilitate the adoption of 3D scanning technologies in resource-limited healthcare settings, enabling their incorporation into standard medical assessments and contributing to greater healthcare services [9].

In response to these challenges, this study aims to assess the reliability of a simplified 3D hand-scanning workflow that utilizes a single, accessible scanning device. Specifically, it aims to develop a structured scanning protocol, implement a concise training intervention for non-specialist users, and assess the impact of training on the quality and consistency of scan outputs.

## 2. Materials and Methods

**Figure 1:**
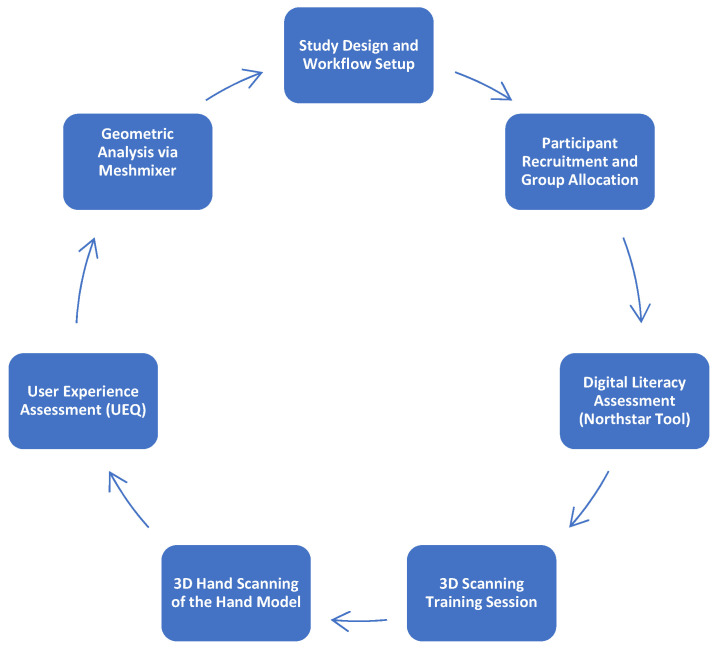
The research protocol followed in this study.

We conducted a cross-sectional comparative study to assess the efficiency of a 3D scanning workflow in capturing hand anatomy. The preparation of the experimental methodology, including the design of the scanning protocol, development of the training content, and 3D printing of the hand replica, was completed in 5 days, the research protocol being shown in Figure 1.

**Figure 2:**
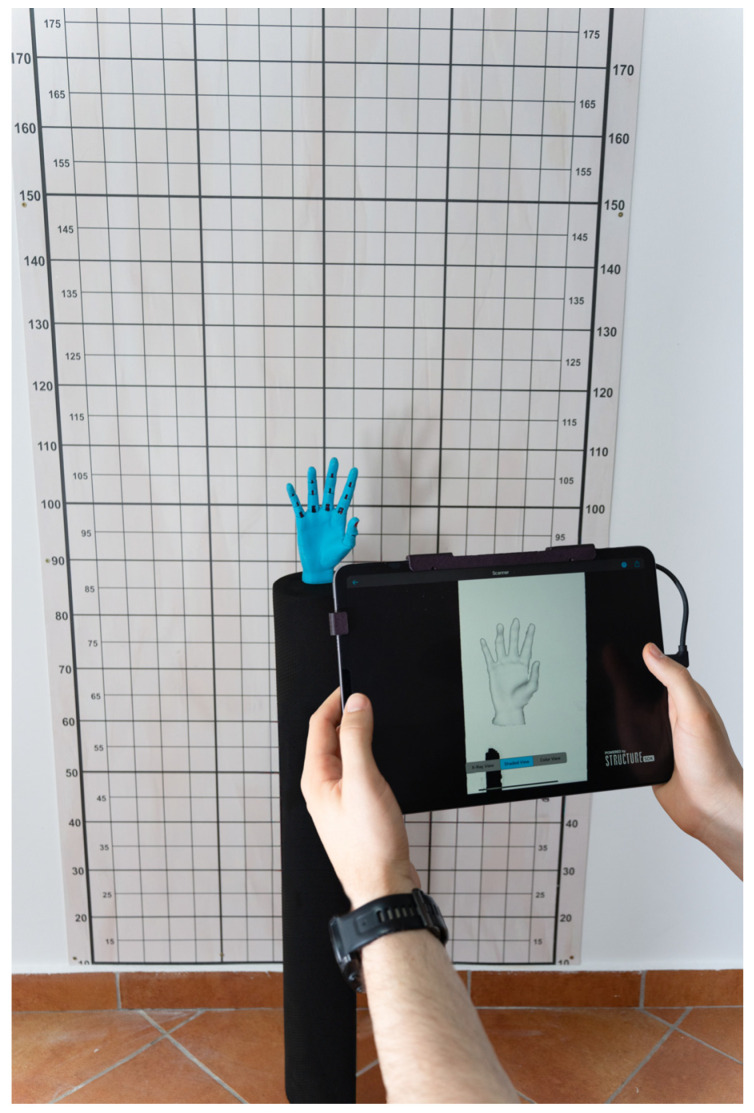
Experimental setup for scanning.

A committee of specialists oversaw participant selection to ensure a consistent methodology and adherence to study criteria, comprising a medical doctor, a physiotherapist, and a specialist in 3D-printed orthoses, each of whom contributed their expertise to different aspects of the study. Participants were recruited from among first-year undergraduate students at the Faculty of Physical Education and Sport, West University of Timisoara, Romania. The inclusion criteria were first-year undergraduate students with no prior knowledge in 3D scanning. Students with advanced knowledge in biomechanics or anatomy and those with a digital or technical background were excluded. Additionally, students in the experimental group who did not fully participate in the training and evaluation sessions were also excluded from the final sample. Following the selection process, 87 students (32 female/55 male) were enrolled in the study. These participants were randomly divided into the following two groups: a control group consisting of 42 students who did not receive any 3D scanning training and an experimental group of 45 students who underwent a structured simplified 15 min 3D scanning training session. The participant enrollment period lasted two weeks, after which the experiment was conducted in a specialized biomechanics laboratory within the faculty. This setting was selected to ensure standardized environmental conditions, controlled lighting, and adequate logistical support for the study, as shown in Figure 2.

The training was designed to provide a fundamental understanding of the scanning process, familiarizing participants with device operation and scanning techniques, thereby establishing a baseline for independent 3D hand scanning. Participants were guided through essential steps, including sensor setup, calibration, optimal hand positioning, and scanning execution, with real-time feedback to help refine their technique, as seen in Appendix A.

**Figure 3:**
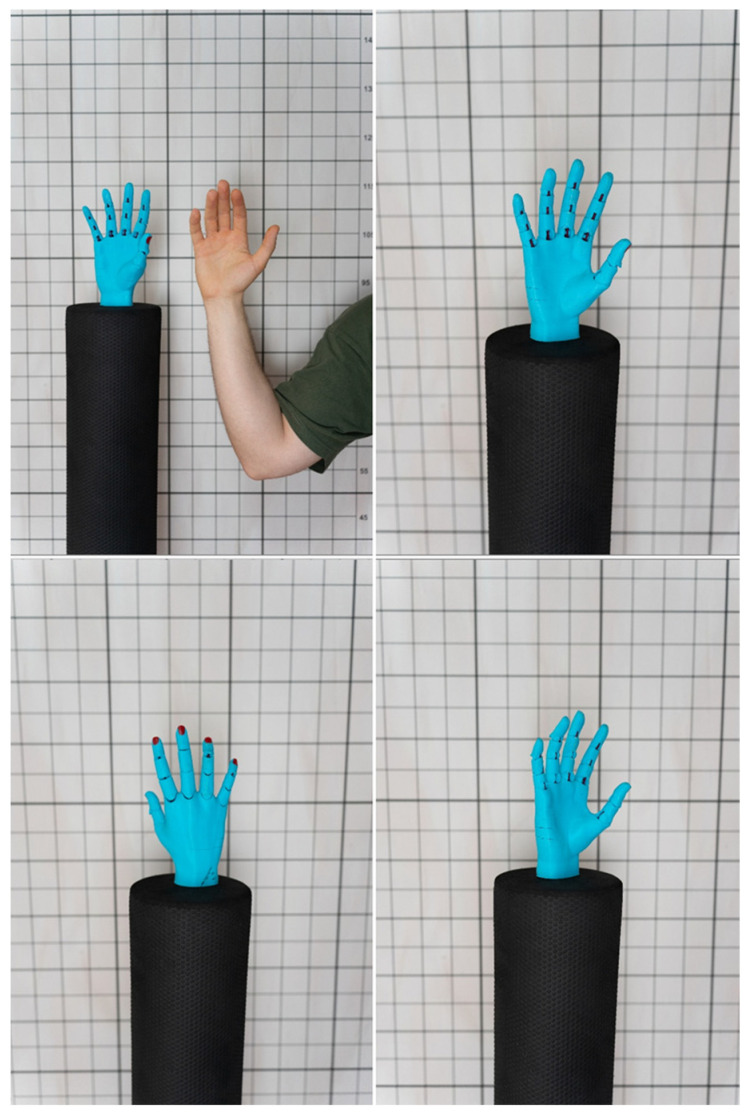
3D-printed replica of a human hand.

A 3D-printed hand model replicating the anatomy of a 19-year-old male subject was introduced to facilitate a more controlled and accurate evaluation, as illustrated in Figure 3 below. Serving as a consistent reference across all groups, it minimized confounding factors, such as subject movement or anatomical variation, ensuring a more objective assessment of scan fidelity.

Each participant completed three scans of the hand replica, resulting in a total of 261 scans, allowing for a direct comparison of scanning precision between trained and untrained users.

The scanning equipment used in the study included the Structure Sensor Pro for iPad (Structure, Boulder, CO, USA), a portable depth-sensing device known for its high resolution and real-time 3D capture capabilities. The sensor featured a resolution of 1280 × 960 pixels, an integrated NU3000 ASIC processor for depth processing and a field of view of 59° × 46°, making it well-suited for capturing the intricate contours of the hand. The scanner operated effectively indoors and outdoors, with a recommended scanning range of 0.3 to 5 m. Scans were performed using an iPad Pro (Apple Inc., Timisoara, Romania; 11-inch, 4th generation), running iPadOS 16.6 (20G75), to ensure compatibility between the scanning hardware and software. The scanning process was carried out using the Structure Sensor Pro’s dedicated application, Scanner, which offers real-time visual feedback to guide the user. This application was used uniformly across all participants to ensure consistency in the scanning procedure.

A structured study protocol was developed to facilitate a comparative evaluation of participant performance, incorporating measurement tools validated in existing literature. The assessment included objective, performance-based tests to evaluate the quality and accuracy of the 3D hand scans and subjective, self-reported measures to capture participants’ experiences with the equipment. To evaluate participants’ digital proficiency, the Northstar Digital Literacy Assessment was used to measure their baseline skills. Subsequently, Meshmixer was utilized to perform geometric analysis and qualitative assessment of the generated 3D models. The User Experience Questionnaire (UEQ) was also administered to evaluate participants’ interaction with the scanning equipment, providing insights into usability, ease of handling, and overall user perception. This dual approach ensured comprehensive analysis, ultimately allowing for a well-rounded evaluation of the scanning process and the training intervention. The Northstar Digital Literacy Tool is a complex assessment designed to evaluate foundational digital competencies for computer and internet navigation. It measures proficiency in the following three key areas: basic computer skills, software applications, and technology use in daily life [10]. In research, Northstar serves as a benchmark for digital literacy, helping identify skill gaps in academic and professional settings [11,12]. It has been utilized to examine disciplinary variations in digital competency, workforce readiness, and the effectiveness of digital education programs. With its structured and standardized evaluation, Northstar offers instant feedback, making it a valuable tool for institutions, educators, and employers seeking to assess digital proficiency [13,14]. Autodesk Meshmixer is a 3D modeling software widely used in fields such as medicine and engineering. It offers a broad set of basic and advanced mesh manipulation tools and is ideal for iterative and exploratory design processes [15]. Integrating generative design techniques further enhances its application in producing complex geometries [16]. Meshmixer enables users to combine and refine mesh elements, which are especially valuable for surgical planning and prosthetic design [17,18]. With integrated analysis and print-preparation features, the software minimizes common 3D printing errors [19]. In medicine, Meshmixer enables the creation of patient-specific anatomical models from CT and MRI data, thereby enhancing surgical visualization and training [20,21]. It has been successfully used in producing 3D-printed models for simulations, such as emergency airway access, and demonstrates substantial accuracy in prosthetic and surgical model design [18,22,23]. As an evaluation tool, Meshmixer has demonstrated measurement consistency with traditional dental assessments and is valuable in complex surgical planning, particularly for modeling congenital heart disease [23,24]. It supports design optimization for patient-specific implants and operates efficiently under computational constraints, aligning with current trends in biomedical engineering and additive manufacturing [17,18,25]. The User Experience Questionnaire (UEQ), developed by Schrepp et al., is a validated tool designed to assess user experience across the following six dimensions: Attractiveness, Efficiency, Perspicuity, Dependability, Stimulation, and Novelty [26,27,28]. Consisting of 26 items, the UEQ enables researchers and developers to systematically capture and quantify user feedback following interaction with digital systems [28,29]. In healthcare-related studies, the UEQ has been widely applied to evaluate mobile health (mHealth) platforms, revealing user priorities, such as dependability and efficiency [30,31,32]. Its broad validation across languages and cultural settings has reinforced the tool’s reliability and supported extended versions, such as UEQ+, to evaluate more profound user experiences [33,34]. Its integration with complementary methods has also established the UEQ as a central resource in human–computer interaction research [35,36].

**Figure 4:**
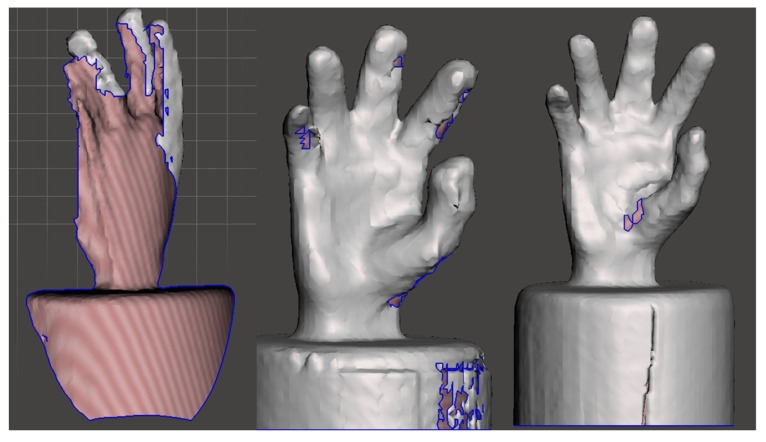
Scans of the hand replica, errors in scanning are indicated in red and outlined with a blue line.

Therefore, participants completed the Northstar Digital Literacy Assessment before any training or scanning activity, allowing for an objective baseline measurement of their digital competencies. The User Experience Questionnaire (UEQ) was administered immediately after the scanning session to capture fresh, subjective feedback on participants’ interaction with the equipment. Conducting both assessments on the same day ensured a consistent timeline, enabling the study to evaluate the influence of digital literacy on scan quality and the immediate user experience following equipment use. However, the Meshmixer evaluation process extended over three weeks, allowing for a detailed scan quality and accuracy analysis. Examples of scans between groups are shown in Figure 4.

The statistical analysis was performed using GraphPad Prism (version 10.0 for Windows). To assess the normality of data distribution, the Shapiro–Wilk test was applied. For comparisons between the control and experimental groups, unpaired *t*-tests were used for normally distributed variables, while the Mann–Whitney U test was applied for non-normally distributed data. This dual approach ensured the appropriate selection of parametric or non-parametric methods, providing a robust evaluation of differences across key outcome measures, including mesh quality parameters and user experience scores. Statistical significance was considered at *p* < 0.05.

## 3. Results

The study’s outcomes are presented below, focusing on statistical significance and descriptive parameters. Group means and standard deviations are reported to complement *p*-values, offering context for the observed differences.

No significant differences were found between groups at baseline regarding age, gender, or digital literacy assessed through Northstar [10], as detailed in Table 1.

The table below presents a comparative analysis of geometric parameters obtained through Meshmixer between the control and experimental groups in the three scans completed. Statistical significance was assessed using appropriate tests, with effect sizes (Cohen’s *d* or correlation coefficient *r*) calculated to evaluate the magnitude of the differences, as seen in Table 2.

Surface Area (m^2^): The experimental group exhibited a significantly larger mean surface area (0.096 ± 0.004 m^2^) compared to the control group (0.09 ± 0.001 m^2^), with *p* < 0.0001 and a large effect size (*d* = 1.6).

Volume (m^3^): Mean volume was significantly greater in the experimental group (0.003 ± 0.0002 m^3^) by comparison to the control group (0.002 ± 0.0004 m^3^), with *p* < 0.001 and a large effect size (*r* = 0.74).

Vertices (number): The number of vertices was significantly higher in the experimental group (14,863 ± 627.1) compared to the control group (13,406 ± 468.6), with *p* < 0.001 and a large effect size (*d* = 2.64).

Triangles (number): Similarly, the experimental group produced models with a significantly greater number of triangles (28,839 ± 1194) compared to the control group (26,955 ± 838.2), having *p* < 0.001 and a large effect size (*d* = 1.84).

Gaps Detected (number): The number of gaps detected in the 3D scans was significantly lower in the experimental group (3.6 ± 1.62) than in the control group (4.08 ± 1.62), with *p* < 0.002 and large effect size (*r* = 0.19).

**Figure 5:**
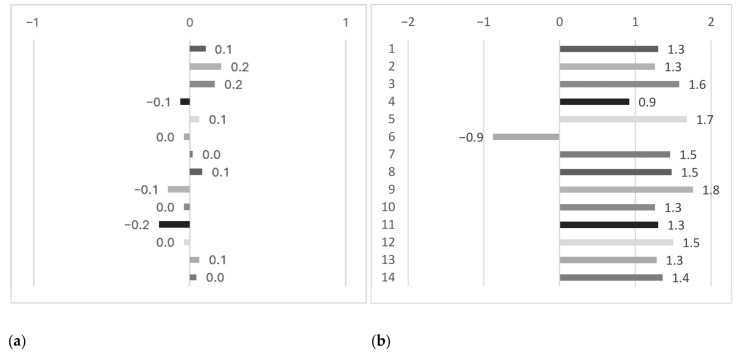
The User Experience Questionnaire (UEQ) showing user perceptions between groups. (**a**) Control group; (**b**) experimental group. Items represent: 1. annoying/enjoyable; 2. not understandable/understandable; 3. boring/exciting; 4. not interesting/interesting; 5. unpredictable/predictable; 6. conventional/inventive; 7. obstructive/supportive; 8. complicated/easy; 9. unlikable/pleasing; 10. usual/leading edge; 11. unpleasant/pleasant; 12. inefficient/efficient; 13. impractical/practical; 14. conservative/innovative.

Figure 5 presents the User Experience Questionnaire (UEQ) results, comparing user perceptions of the two evaluated groups in this study. In the control group (left panel), responses were found to be concentrated around neutral or slightly negative values. Dimensions such as pleasantness, innovation, and supportiveness received notably low scores, suggesting a generally underwhelming user experience. No dimensions showed strongly positive ratings, indicating that the control condition was perceived as neither particularly engaging nor effective. In contrast, the experimental group (right panel) received consistently positive evaluations across almost all dimensions. The 3D scanner was rated particularly highly in excitement, ease of use, supportiveness, pleasantness, and practicality, indicating a strong overall user experience. These ratings suggest that participants in the trained group experienced fewer usability issues, such as frustration or confusion, and that the short training session effectively enhanced both confidence and comfort during the task.

## 4. Discussion

### 4.1. 3D Scanning of the Hand

Studies investigating the reliability of two standard techniques, laser, and structured-light scanning demonstrated that both are significantly influenced by environmental conditions and device specifications [37]. Other research introduced a depth-camera-based approach designed for scanning the upper limb, emphasizing non-contact methods as both accurate and more comfortable for clinical use. Their work highlights an encouraging trend toward access to high-quality scanning [38]. Other studies showed how scanning conditions affect outcomes, albeit in facial applications, suggesting transferable insights into the hand’s equally complex structure [39]. Some studies in the field have explored how these technologies influence the design of customized orthotic solutions, underscoring their importance in delivering individualized care [6]. The scan quality found in the experimental group from our study aligns with prior research, showing that simplified, contact-free scanning methods can capture complex anatomical structures with clinically acceptable accuracy. These findings support integrating accessible 3D scanning technologies into clinical workflows, particularly for applications such as orthotic design where precision is critical.

### 4.2. Non-Professionals in 3D Scanning

It has been demonstrated that depth-camera-based systems and structured-light scanners can yield accurate anatomical models even when operated by users without formal training, highlighting a shift toward more accessible, user-friendly scanning tools [37,38]. Improvements in software and apps have simplified the scanning process, enhancing usability for casual users. Research supports the effectiveness of low-cost handheld devices for producing orthopedic models, emphasizing their accuracy and practicality in non-expert hands [40]. Although challenges such as motion artifacts persist, especially in facial scanning, advancements in algorithms continue to mitigate these issues [6]. Other studies have shown that even basic tools can yield valid anthropometric data, demonstrating how data collection can be simplified for broader use [41]. Research has also confirmed that depth cameras provide intuitive interfaces, enabling reliable scans with minimal training [42,43]. Our findings align with and extend this research by demonstrating that minimally trained users can produce high-quality hand scans when guided by a structured workflow. This reinforces the practical value of accessible 3D scanning systems in clinical and rehabilitative settings, where operator expertise may vary.

### 4.3. Structure Sensor Pro and Its Uses in Medical and Research Fields

The Structure Sensor Pro has become a significant innovation in 3D scanning, particularly valued in medical and research settings. Its rapid capturing of high-resolution anatomical data makes it ideal for patient-specific modeling and therapeutic planning [41]. Studies have demonstrated its effectiveness in producing customized prosthetics that closely match individual anatomical structures, improving treatment outcomes [43]. Other work evaluated its role in developing wrist splints through anatomical data customization, emphasizing its relevance in rehabilitation [44]. The sensor also supports accurate gait analysis, with performance comparable to gold-standard methods, providing essential data for evaluating mobility impairments [45]. Additionally, its adaptability allows it to be used in various clinical applications, from orthotic assessments to surgical planning and monitoring, making it a versatile tool in research and clinical practice [46]. Consistent with previous literature, this study utilized the Structure Sensor Pro for hand geometry acquisition and verified its reliability in generating high-resolution anatomical models. Its performance within a structured protocol reinforces its relevance for clinical applications.

### 4.4. Comparative Studies of 3D Scanning

Research findings have demonstrated improvements in 3D reconstruction accuracy using depth cameras, marking a shift from conventional to modern sensor-based approaches [42]. Similarly, the utility of combining 3D scanning with printing was demonstrated in creating personalized wrist orthoses, thereby reinforcing the value of integrating these tools into routine clinical workflows [47]. Moreover, several systematic reviews and meta-analyses found that portable face-scanning systems can achieve accuracy comparable to stationary systems, with mean discrepancies under 1.0 mm, meeting clinical standards. Among portable methods, no significant differences in accuracy were found across technologies. Stereophotogrammetry excels in capturing detailed surface textures like skin features but is highly sensitive to light conditions and camera settings. Laser and structured-light scanners offer better control over lighting and faster single-scan capture; although, reflective or transparent surfaces can interfere with accuracy [40]. Another research in the field stressed the importance of understanding the limitations and differences between full-body and partial scans, particularly when applied to health and ergonomic assessments. Laser line systems offer high accuracy for full-body scans but are costly and less portable. Structured-light scanners and stereophotogrammetry are better suited for partial-body applications, offering faster capture and finer surface detail, which is ideal for clinical use. Millimeter wave scanners enable clothed full-body scans quickly but lack the precision needed for detailed anatomical modeling [48]. This research demonstrates the effectiveness of structured-light scanning for partial-body applications, specifically in capturing hand geometry. Unlike previous research that focused on comparing different scanning technologies, this study offers a complementary perspective by evaluating the same technology with different user groups. This approach reveals how operator experience and training influence scan quality, adding a new dimension to the discussion on usability and accuracy. The results confirm that, when paired with a streamlined workflow, even minimally trained users can produce clinically acceptable anatomical models, reinforcing the potential of these technologies for broader integration into digital rehabilitation.

### 4.5. 3D Scanning Training

Prior research introduced a structured framework for body measurement using 3D scanning, outlining key phases such as preparation, scanning, feature extraction, model fitting, and measurement extraction. By segmenting the process, the authors present a compelling case for how practitioners across varying levels of expertise can effectively engage with 3D scanning techniques [5]. The integration of 3D scanning within digital workflows for prosthetics and orthotics has been also explored in clinical settings, where it was demonstrated how these technologies can streamline the preparation of prosthodontic devices. By automating the capture of detailed surface data, 3D scanning significantly reduces the need for manual measurements and associated errors. This highlights the importance of implementing training initiatives that equip clinicians with the necessary digital skills to confidently adopt these workflows [43]. Portnoy et al. [49] explored the integration of 3D scanning into occupational therapy (OT) education by examining how OT students prepared finger orthoses using manual and automated 3D printing methods. A total of 36 undergraduate OT students, all of whom had completed approximately 10 h of formal training, participated in the study. While the students were familiar with manual fabrication techniques, they had no prior experience with 3D printing or the software used in the study. To bridge this gap, a brief 5 min instructional session was provided to students before they engaged with the digital tools. This training included using a digital caliper to collect and input five anatomical measurements into the software, which then generated a patient-specific, printable STL file. Despite their inexperience with 3D technology, students expressed greater satisfaction with the 3D-printed orthoses, particularly regarding fit, aesthetics, and overall product quality. Although the manual method was faster, likely due to prior exposure and practice, the 3D method was favored by the majority for more complex orthotic designs. This finding suggests that with minimal training, OT students can effectively engage with digital fabrication tools and recognize their clinical potential [49]. Thus, building on prior research, this study demonstrates the usability of 3D scanning and its approachable nature for inexperienced users. By engaging students in the scanning process, we were able to observe how quickly foundational skills can be developed and applied to clinically relevant tasks. Rather than focusing solely on technological performance, this approach highlights training as a key enabler for successful implementation.

### 4.6. 3D Scanning and AI

Modern 3D scanning technologies provide highly accurate anatomical data, forming the foundation for AI-driven processes in orthotic design. The level of detail captured through these techniques supports the development of applications capable of generating personalized orthotic solutions based on an individual’s biomechanics and rehabilitation goals [50]. Once a patient’s 3D segment model is created, AI algorithms are applied to streamline the design of orthotic and prosthetic devices. In prosthetics, for instance, machine learning models can process data such as gait patterns to produce devices that adapt dynamically to users’ movements [51]. This adaptive functionality enhances user comfort and effectiveness, offering more natural movement than conventional, rigid designs [52]. Together, these innovations are transforming how orthopedic devices are conceptualized and manufactured. Moreover, AI facilitates a more tailored and anticipatory approach to orthotic design. Traditional methods often involve significant adjustments after production to ensure proper fit and function [53]. In contrast, AI allows for predicting and integrating patient-specific structural and functional requirements early in the design process. This proactive approach reduces time-consuming refinements and supports a smoother, more individualized rehabilitation experience [54]. Even as software and AI tools grow more sophisticated, scanning remains the foundation of digital workflows. High-quality scan data enables later processes to deliver meaningful, patient-specific outcomes. This study focused on the scanning process, emphasizing its importance and accessibility. When accurate anatomical data is captured from the start, it lays the groundwork for practical evaluation, diagnosis, and even orthotic manufacturing.

### 4.7. Implications and Context

This study examined the influence of user experience and training on 3D scan quality when using a single, low-cost device, allowing for a focused evaluation of operator-related variability. By applying the same scanning equipment across differently trained group, including participants with minimal technical backgrounds, the study highlights the practical value of a structured, time-efficient training protocol. This approach contributes to ongoing discussions around usability, accessibility, and user-centered workflow design in 3D scanning.

The integration of objective mesh quality metrics with subjective user feedback offers a multidimensional perspective that may support future research on training conditions for students, healthcare professionals, or researchers in the field. These findings are particularly relevant for clinical and educational contexts where streamlined procedures and limited digital expertise are common. Moreover, the results underscore the potential for developing scalable training models that support the broader adoption of digital health technologies and remote anatomical data acquisition.

## 5. Conclusions

This study demonstrates the feasibility and effectiveness of a simplified 3D hand-scanning workflow that requires minimal training and utilizes accessible and commercial technology. By combining objective scan analysis with digital literacy and user experience assessment, the research highlights how structured guidance enables non-specialists to perform accurate scans of complex human anatomy. These findings support the broader integration of 3D scanning into clinical, educational, and rehabilitation settings, reinforcing its potential as a low-cost, user-friendly tool for personalized healthcare and digital innovation.

## Figures and Tables

**Table 1:** Baseline characteristics of the study groups.

	Control Group (n = 42)	Experimental Group (n = 45)	*p*-Value (*t*-Test)
Age (mean ± SD)	19.9 ± 1.1	19.8 ± 1.07	*p* = 0.72
Gender f/m (n; %)	14/28; 34/66	18/27; 41/59	*p* = 0.52
Digital competence (%)	87.5% ± 36.27	88.3% ± 29.04	*p* = 0.22

**Table 2:** Comparison of scanning parameters between the study groups.

Parameters	Control Group (mean ± SD)	Experimental Group (mean ± SD)	*p*-Value	Size Effect
Surface area (m^2^)	0.09 ± 0.001	0.096 ± 0.004	*p* < 0.0001	*d* = 1.6
Volume (m^3^)	0.002 ± 0.0004	0.003 ± 0.0002	*p* < 0.0001	*r* = 0.74
Vertices (nr.)	13,406 ± 468.6	14,863 ± 627.1	*p* < 0.0001	*d* = 2.64
Triangles (nr.)	26,955 ± 838.2	28,839 ± 1194	*p* < 0.0001	*d* = 1.84
Gaps detected (nr.)	4.09 ± 1.62	3.6 ± 1.62	*p* < 0.002	*r* = 0.19

## Data Availability

The original contributions presented in this study are included in the article. Further inquiries can be directed to the corresponding author.

## Appendix A

### Training Overview

A standardized 16 min training session was developed and delivered to participants in the experimental group. The training took place in a controlled laboratory environment equipped with standardized lighting, minimal background interference, and sufficient space for maneuvering around the subject during scanning. Each participant had access to identical hardware, including an iPad paired with the Structure Sensor Pro, as well as all necessary mounting equipment and calibration tools.

The session was designed to develop baseline operational competency in using the scanning system, with particular emphasis on capturing hand anatomy. Instruction focused on essential procedures, such as device assembly, sensor calibration, subject positioning, real-time scan monitoring, and 3D model verification.

Given that all participants had no prior experience with 3D scanning, the content was intentionally concise and practically oriented. By standardizing the environment and instructional content, the study sought to isolate the effect of structured training on scan quality outcomes.

The following components were covered during the training:

- Introduction (1 min):

The session began with a brief overview of the training objectives, the role of 3D scanning in the study, and participant expectations. This context helped frame the importance of mastering the scanning procedure.

Step 1: Hardware Familiarization (2 min):

Participants were introduced to the Structure Sensor Pro and the iPad tablet, including accessories such as mounts and charging cables. Basic handling, safety considerations, and device care tips were also discussed.

Step 2: Equipment Check (1 min):

A quick demonstration was provided on connecting the sensor to its power source and checking the battery levels for both the sensor and iPad, ensuring smooth operation throughout the scanning session.

Step 3: Sensor Mounting and Calibration (2 min):

Attendees observed how to securely attach the sensor to the tablet and adequately calibrate the device using the designated application. This step emphasized the importance of calibration in achieving accurate scan results.

Step 4: Application Setup and Mode Selection (2 min):

The trainer guided participants through launching the scanning app, establishing a connection with the sensor and selecting the appropriate scanning mode—specifically, the hand segment mode—while explaining the purpose of other available modes.

Step 5: Positioning and Environment Setup (2 min):

Instructions were given on placing the subject at the center of the scanning space, maintaining a proper distance (0.3–5 m) and positioning the hand for optimal scanning. The importance of a clutter-free environment and user mobility around the subject was also addressed.

Step 6: Live Scanning Demonstration (2 min):

A practical, real-time demonstration of the scanning process was carried out. Participants observed how to follow the on-screen feedback provided by the app to capture a complete and detailed scan.

Step 7: Review and Saving (1 min):

The significance of verifying the scan’s completeness was emphasized. Participants were shown how to review the model for missing details or distortions and save it properly within the device.

Step 8: Post-Processing Overview (Optional-1 min):

A brief explanation of post-processing techniques was provided, including refining scans and preparing them for other applications, such as modeling or printing.

- Q&A and Conclusion (2 min):

Participants were invited to ask questions, share their observations, or seek clarification on any part of the training. The session concluded with a recap of the key takeaways and a reminder of the importance of consistent practice to enhance scanning proficiency.
